# Sulforaphane Attenuates Contrast-Induced Nephropathy in Rats via Nrf2/HO-1 Pathway

**DOI:** 10.1155/2016/9825623

**Published:** 2016-02-24

**Authors:** Zhihong Zhao, Guixiang Liao, Qin Zhou, Daoyuan Lv, Harry Holthfer, Hequn Zou

**Affiliations:** ^1^Department of Nephrology, The Third Affiliated Hospital of Southern Medical University, Guangzhou, Guangdong 510630, China; ^2^Department of Radiation Oncology, Shenzhen People's Hospital, Second Clinical Medicine College of Jinan University, Shenzhen, Guangdong 518020, China; ^3^National Centre for Sensor Research/BioAnalytical Sciences, Dublin City University, Dublin 9, Ireland

## Abstract

*Background*. Oxidative stress plays an important role in the pathogenesis of contrast-induced nephropathy (CIN). The aim of this study was to investigate the antioxidant effects of sulforaphane (SFN) in a rat model of CIN and a cell model of oxidative stress in HK2 cells.* Methods*. Rats were randomized into four groups (*n* = 6 per group): control group, Ioversol group (Ioversol-induced CIN), Ioversol + SFN group (CIN rats pretreated with SFN), and SFN group (rats treated with SFN). Renal function tests, malondialdehyde (MDA), and reactive oxygen species (ROS) were measured. Western blot, real-time polymerase chain reaction analysis, and immunohistochemical analysis were performed for nuclear factor erythroid-derived 2-like 2 (Nrf2) and heme oxygenase-1 (HO-1) detection.* Results*. Serum blood urea nitrogen (BUN), creatinine, and renal tissue MDA were increased after contrast exposure. Serum BUN, creatinine, and renal tissue MDA were decreased in the Ioversol + SFN group as compared with those in the Ioversol group. SFN increased the expression of Nrf2 and HO-1 in CIN rats and in Ioversol-induced injury HK2 cells. SFN increased cell viability and attenuated ROS level in vitro.* Conclusions*. SFN attenuates experimental CIN in vitro and in vivo. This effect is suggested to activate the Nrf2 antioxidant defenses pathway.

## 1. Introduction

Contrast-induced nephropathy (CIN) is an important complication in diagnostic and interventional procedures that requires the use of iodinated contrast media [1]. CIN is generally defined as an otherwise unexplained acute impairment in renal function, manifested as serum creatinine increases of 0.5 mg/dL or more than 25% after the administration of contrast media [1]. CIN is the third most common cause of acute kidney injury in inpatient settings, accounting for 10–25% of all acute kidney injury cases, and nearly 150,000 patients are estimated to develop CIN each year worldwide [2, 3].

CIN may increase the incidence of in-hospital morbidity and mortality, increase the costs of medical care, and prolong hospital stays [4]. Despite the advent of advanced contrast media and improvements in preventive strategies, the prevention of CIN remains challenging, and new and effective strategies for the prevention of CIN are urgently needed.

The mechanism of CIN is poorly understood but may include the direct tubular toxicity of contrast media [1, 5, 6] and the production of excessive levels of reactive oxygen species (ROS) [7]. A growing body of evidence indicates that ROS may play a critical role in the pathophysiology of CIN [8–10]. Accordingly, several studies have reported that several potent scavenging compounds can effectively prevent CIN [7, 11–13].

Sulforaphane (SFN) is abundant in cruciferous vegetables and is a potential antioxidant [14]. Moreover, it is a new promising agent for the prevention of a range of diseases. Specifically, several studies have reported that the protective properties of SFN against diseases may involve the Kelch-like ECH-associated protein-1 (Keap1)/nuclear factor erythroid-derived 2-like 2 (Nrf2) antioxidant response element (ARE) signaling pathways. SFN, a widely used Nrf2 activator, exhibits protective properties in experimental diabetic nephropathy [15], renal reperfusion injury [16], lupus nephritis [17], and renal fibrosis [18].

Nevertheless, the antioxidant effect of SFN in the context of renal injury induced by the administration of contrast media has not been previously investigated. Therefore, the present study sought to assess the antioxidant effects of SFN in an experimental model of CIN in rats and HK2 cells.

## 2. Materials and Methods

### 2.1. Animals and Groups

The animals were cared for according to the guidelines of Southern Medical University for the care and use of laboratory animals. Adult Sprague Dawley rats purchased from the animal center of Southern Medical University (180–200 g) were fed a standard rat chow diet and tap water and housed in individual cages under controlled conditions of light (12 h/12 h light/dark cycle) and temperature (24 ± 2°C). The rats were acclimatized for one week before the experiment.

The rats were randomly divided into four groups of 6 rats each as follows: control group (control), Ioversol group, Ioversol + SFN group, and SFN group.

### 2.2. CIN in Rats

CIN was induced according to a previously detailed CIN protocol [1, 7, 19]. Briefly, the rats were anesthetized using an intraperitoneal (i.p.) injection of pentobarbital sodium, and drugs were then administered via the tail vein. The administered drugs consisted of indomethacin at a dose of 10 mg/kg, followed by Nw-nitro-L-Arginine methyl ester (L-NAME) at a dose of 100 mg/kg and Ioversol (8.3 mL Ioversol/kg, 2.9 g/kg organically bound iodine) 15 and 30 min later.

### 2.3. Study Protocol

Control group (*n* = 6): rats received 0.9% saline injection through the tail vein.

Ioversol group (*n* = 6): CIN was induced and no additional treatment was given.

Ioversol + SFN group (*n* = 6): rats were administrated SFN at a dose of 5 mg/kg for consecutive 5 days before establishing CIN.

SFN groups (*n* = 6): rats were administrated SFN at a dose of 5 mg/kg for consecutive 5 days and then 0.9% saline injection followed through the tail vein.

### 2.4. Histological Examination of Renal Tissues

After a total of 72 h after the administration of ionic high-osmolar Ioversol or saline, the animals were anesthetized with i.p. injections of pentobarbital sodium (60 mg/kg). Both kidneys were harvested, cut into four equatorial sections, and immediately washed with cold phosphate-buffered saline (PBS). Two pieces of kidney were fixed in 4% formalin for the histopathological examination (hematoxylin and eosin, H&E) and immunohistochemistry. The following antibodies were used: rabbit polyclonal anti-Nrf2 (Abcam, Cambridge, MA), rabbit polyclonal anti-HO-1 (Abcam), and rabbit polyclonal anti-NAD(P)H: quinone oxidoreductase 1 (NQO-1). Paraffin-embedded tissues were gradually deparaffinized and subjected to antigen retrieval by microwaving in 0.01 M sodium citrate buffer at pH 6.0. Following microwave treatment, the slides were incubated with the rabbit anti-mouse Nrf2 antibody (Abcam) at 4°C overnight. Endogenous peroxidase was inactivated by incubation in 0.3% hydrogen peroxide in methanol, and endogenous biotin was blocked using a streptavidin-biotin blocking system (Golden Bridge Biotechnology Co., China). The antibody reaction products were observed with the chromogen 30-diaminobenzidine tetrachloride (DAB). After a final wash in distilled water, the sections were counterstained with hematoxylin, dehydrated, cleared, and mounted. The remaining tissue sections were flash-frozen in liquid nitrogen and stored at −80°C until use in the real-time polymerase chain reaction (PCR) and western blot analyses.

### 2.5. Biochemical Analysis

The blood obtained by intracardiac puncture was centrifuged for biochemical analysis. Rat serum samples were used for evaluation of blood urea nitrogen, as well as creatinine with an automatic biochemical analyzer (AU5400, Olympus, Tokyo, Japan).

### 2.6. Real-Time PCR Analysis

Total RNA was extracted from renal tissue and HK2 cells with TRIZOL (Takala, Dalian China) and the mRNA were used to synthesize cDNA using the Transcriptor First Strand cDNA synthesis kit (Takala, Dalian, China). Primers used for gene amplification are showed in Table 1.

### 2.7. Oxidant Parameters Measurement

The levels of superoxide dismutase (SOD) and MDA were assessed using two commercial detection kits (Nanjing Jiancheng Bioengineering Institute) according to the manufacturer's protocol and a previous study [20].

HK2 cells (ATCC, USA) were cultured in K-SFM at 37°C and 5% CO_2_. HK2 cells were treated with H_2_O_2_ (500 *μ*mol/L) or 50 mg/mL Ioversol for 24 h to induce oxidative injuries. To assess the ability of SFN to protect cells from injury, HK2 cells were preincubated with 5 *μ*Mol/L SFN 30 min before treatment with H_2_O_2_ or 50 mg/mL Ioversol. The intracellular ROS were then measured by detecting the oxidative conversion of 2′,7′-dichlorofluorescein diacetate (DCFH-DA) (Sigma, USA) to fluorescent dichlorofluorescein (DCF) in permeable cells. Cell death was quantified using the 3-[4,5-dimethylthiazol-2-yl]-2,5-diphenyltetrazolium bromide (MTT) assay.

### 2.8. Nrf2 Knockdown by siRNA Transfection

Scrambled small-interfering RNA (siRNA) and Nrf2 siRNA were purchased from RiboBio Co. After the cells were seeded in six-well plates, they were transfected using Lipofectamine 2000 (Invitrogen) according to the manufacturer's instructions. The transfected cells were subjected to experiments 48 h after transfection.

### 2.9. Western Blot Analysis

For the immunoblot analysis, the frozen kidney tissue and HK2 cells were homogenized in lysis buffer [0.1 mol/L Tris buffer (pH 7.4), 0.1 mmol/L EDTA] in the presence of 1 mmol/L dithiothreitol, 1 mmol/L phenylmethylsulfonyl fluoride, and a protease inhibitor cocktail (Roche, USA). The protein concentrations of the samples were measured using the bicinchoninic acid method. Equivalent amounts of cell lysates were electrophoresed, transferred to nitrocellulose membranes, and incubated with primary antibodies overnight at 4°C. After blocking with 5% skim milk, the membranes were incubated with anti-Nrf2, anti-NQO-1, or anti-HO-1 at a dilution of 1 : 1000.

After washing, the membranes were incubated with a secondary antibody conjugated with horseradish peroxidase for 1 h at room temperature. Immunoreactive bands were visualized by using the SuperSignal West Pico enhanced chemiluminescent substrate (ECL, Pierce, IL, USA). The bands were quantified by densitometry using GeneTools from Syngene.

### 2.10. Statistical Analysis

The results are presented as the means ± standard error (SE). Student's *t*-test or one-way analysis of variance (ANOVA) was used to determine the significance of differences in multiple group comparisons using the SPSS software 13.0.

## 3. Results

### 3.1. SFN Prevents CIN-Associated Renal Dysfunction in CIN Rats

**Figure 1:**
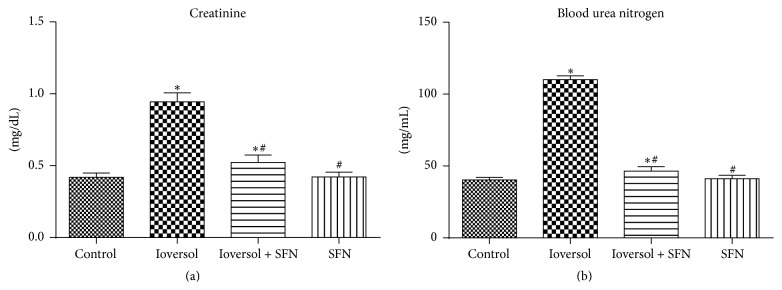
SFN decreased the levels of serum creatinine (a) and blood urea nitrogen (b) in CIN rats. ^*∗*^ *P* < 0.05 versus control group; ^#^ *P* < 0.05 versus Ioversol group.

The serum BUN and creatinine levels in each group are shown in Figure 1. Both the serum BUN and creatinine levels were significantly increased in the Ioversol group compared with the control group (*P* < 0.05). The results were consistent with those of previous studies demonstrating that Ioversol causes renal dysfunction [7, 21, 22]. In the Ioversol + SFN group, the administration of SFN significantly decreased the serum BUN and creatinine levels compared with those in the Ioversol group (*P* < 0.05), which indicated that SFN may play a renoprotective role.

### 3.2. SFN Ameliorated Renal Histological Damage

**Figure 2:**
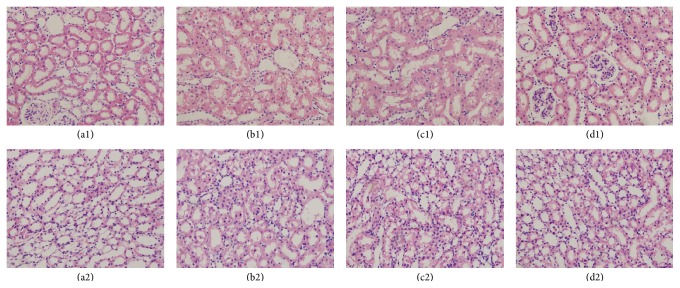
Representative histologic samples from different groups, magnification ×400. Control group (a1, a2); Ioversol group (b1, b2); Ioversol + SFN group (c1, c2); and SFN group (d1, d2).

The pathological findings of kidney sections in all groups are shown in Figure 2. The kidney sections of the control group animals did not exhibit marked histological changes. The kidney sections of the Ioversol group animals exhibited severe damage, consisting of lesions, tubular necrosis, and hemorrhagic casts. In the Ioversol + SFN group, pretreatment with SFN significantly attenuated the development of these lesions and tissue damage. These kidney pathological findings suggested that SFN may protect CIN rats from renal histological damage (Figure 2).

**Figure 3:**
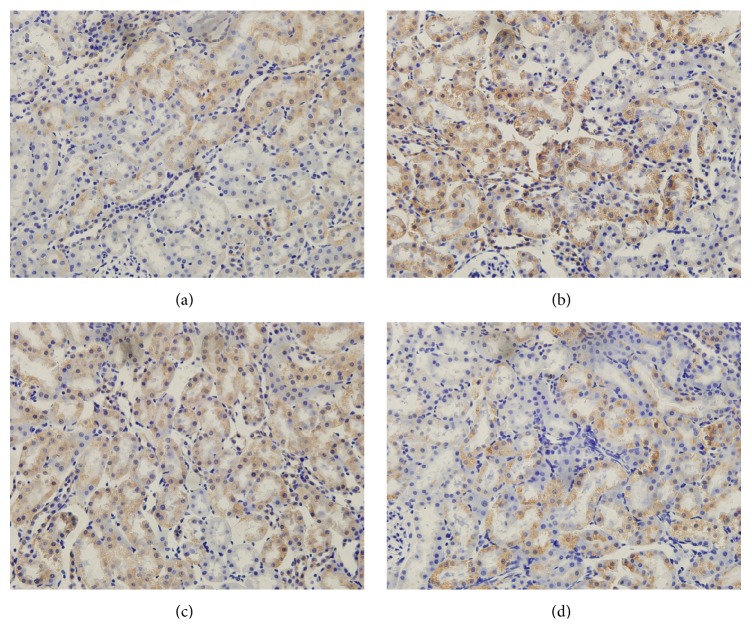
Immunohistochemical photograph of Nrf2 in the kidneys of different groups. Original magnification ×400. (a) Control group; (b) Ioversol group; (c) Ioversol + SFN group; and (d) SFN group.

**Figure 4:**
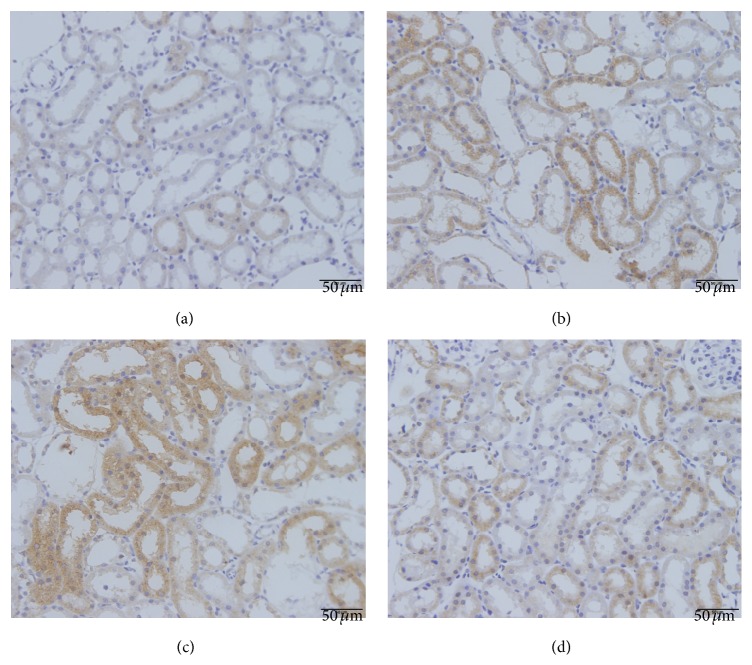
Immunohistochemical photograph HO-1 in the kidneys of different groups. Original magnification ×400. (a) Control group; (b) Ioversol group; (c) Ioversol + SFN group; and (d) SFN group.

**Figure 5:**
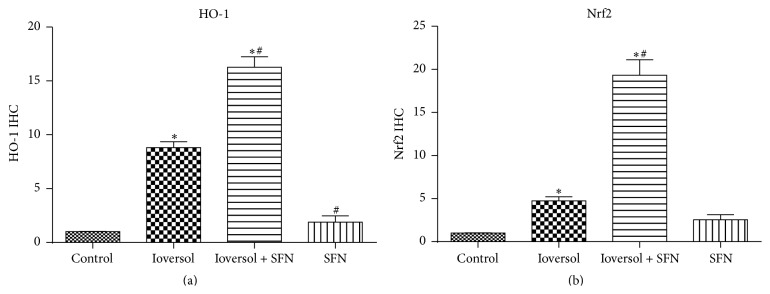
Semiquantitative analysis of Nrf2 and HO-1 immunoactivities in the kidneys of different groups. (a) Nrf2, (b) HO-1. Data are presented as the means ± SE (*n* = 6). ^*∗*^ *P* < 0.05 versus control group; ^#^ *P* < 0.05 versus Ioversol group.

The Nrf2 immunohistochemistry results are shown in Figure 3. Nrf2 immunopositivity was especially marked in the glomeruli and tubular epithelium. Strong positive Nrf2 staining was detected in CIN rats treated with SFN (Figure 3(c)). Due to severe oxidative stress in CIN rats, Nrf2/HO-1 signaling was significantly activated (*P* < 0.05, Ioversol versus control group). The HO-1 immunohistochemistry results are shown in Figure 4. The strongest HO-1 immunopositivity was observed in the Ioversol + SFN group (Figure 4(c)). HO-1-positive staining was also observed in the Ioversol group. The results of a semiquantitative analysis of the Nrf2 and HO-1 immunoactivities in the kidneys of different groups are shown in Figure 5.

### 3.3. SFN Attenuated MDA Levels and Increased SOD Levels in Renal Tissues

**Figure 6:**
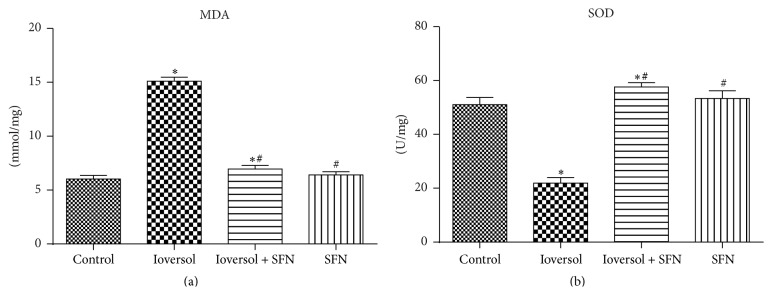
SFN decreased the levels of MDA (a) and increased the levels of SOD (b) in CIN rats. ^*∗*^ *P* < 0.05 versus control group; ^#^ *P* < 0.05 versus Ioversol group.

The lipid peroxidation product MDA was analyzed as an index of oxidative stress. The renal tissue MDA levels in each group increased, as shown in Figure 6(a). The levels of MDA in the renal tissue in the Ioversol group were significantly higher than those in the control group (*P* < 0.05). SFN significantly attenuated these increases in the MDA levels in the Ioversol + SFN group, and the MDA levels did not significantly differ between the control and Ioversol + SFN groups. Moreover, contrast media decreased the SOD activities, but this effect was inhibited by pretreatment with SFN (Figure 6(b)).

### 3.4. SFN Pretreatment Enhances Nrf2 Target Gene Expression

**Figure 7:**
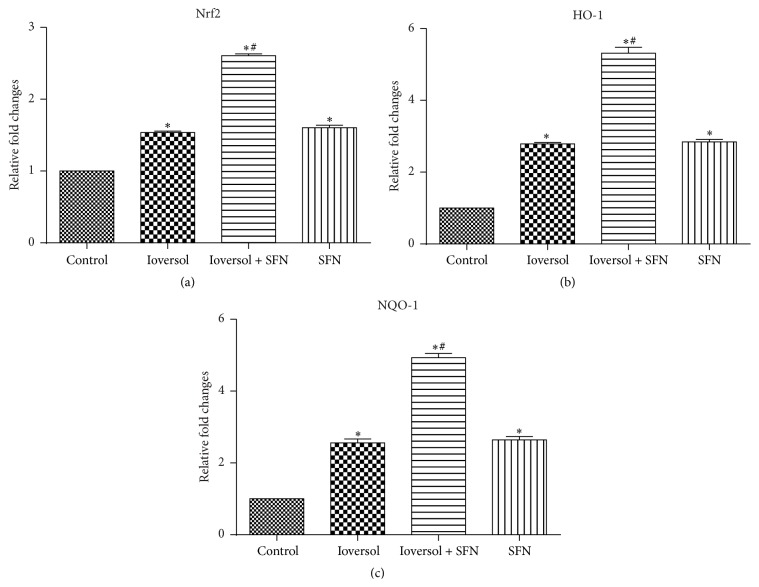
SFN increased the expression levels of Nrf2, HO-1, and NQO-1 in CIN rats. (a) The relative expression of Nrf2; (b) the relative expression of HO-1; and (c) the relative expression of NQO-1. ^*∗*^ *P* < 0.05 versus control group; ^#^ *P* < 0.05 versus Ioversol group.

SFN treatment has been previously shown to increase the expression of Nrf2 and its downstream genes (e.g., HO-1 and NQO-1) [15, 17, 18]. To examine whether the renoprotective role of SFN in contrast-induced renal toxicity is associated with Nrf2 activation, the mRNA expression levels of Nrf2 and its target genes, NQO-1 and HO-1, were detected using real-time PCR. As demonstrated in Figure 7, the administration of SFN significantly increased the gene expression of Nrf2, NQO-1, and HO-1. The results also indicated that SFN protected the kidney from contrast-induced injury via the Nrf2/HO-1 pathway.

### 3.5. SFN Pretreatment Enhances Nrf2 Nuclear Translocation and Increases NQO-1 and HO-1 Protein Expression

**Figure 8:**
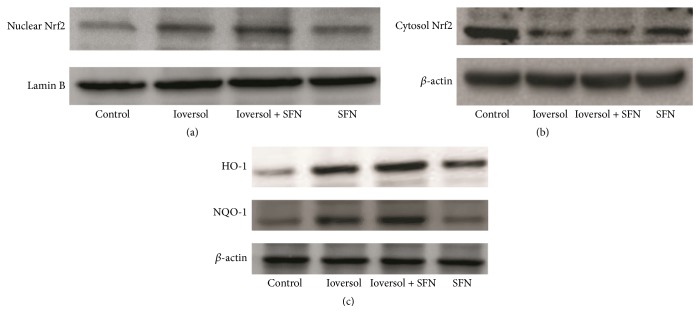
The protein levels in different groups. (a) SFN pretreatment enhanced Nrf2 nuclear translocation. (b) Nrf2 protein levels in the cytoplasm. (c) SFN pretreatment increased NQO-1 and HO-1 protein levels in CIN rats.

To confirm that SFN treatment activates the Nrf2/HO-1 pathway, the protein levels of Nrf2, HO-1, and NQO-1 were measured by western blot analysis. As shown in Figure 8, SFN treatment significantly increased Nrf2 nuclear translocation (Figure 8(a)). Moreover, SFN treatment also increased the HO-1 and NQO-1 protein levels (Figure 8(c)).

### 3.6. SFN Protects against Oxidative Stress and Increased Cell Viability In Vitro

**Figure 9:**
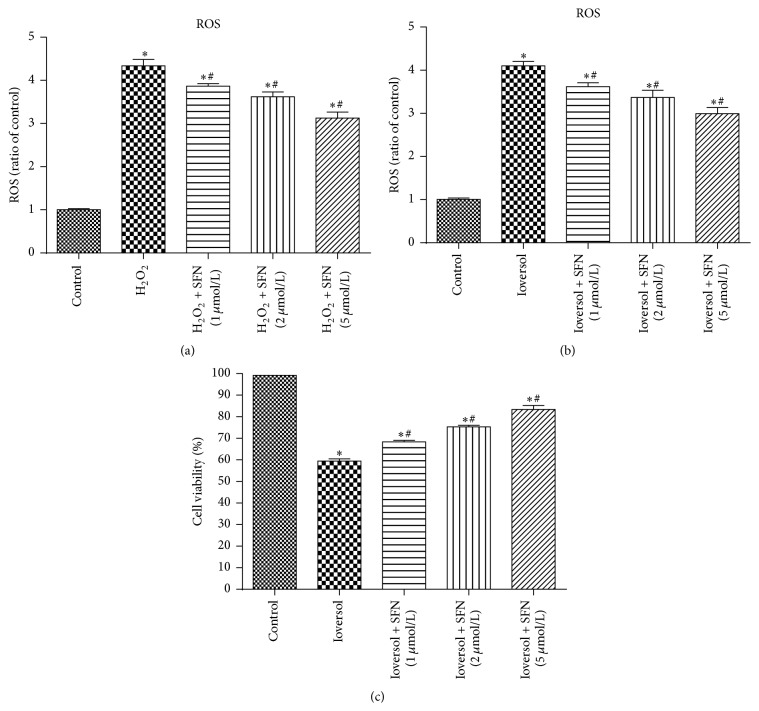
SFN protected against H_2_O_2_- or Ioversol-induced injury in HK2 cells. (a) SFN protected against H_2_O_2_-induced reactive oxygen species. ^*∗*^ *P* < 0.05, versus control group; ^#^ *P* < 0.05, versus H_2_O_2_ group. (b) SFN protected against Ioversol-induced reactive oxygen species, ^*∗*^ *P* < 0.05, versus control group; ^#^ *P* < 0.05, versus Ioversol group. (c) SFN increased cell viability in Ioversol-induced injury cells, ^*∗*^ *P* < 0.05, versus control group; ^#^ *P* < 0.05, versus Ioversol group.

**Figure 10:**
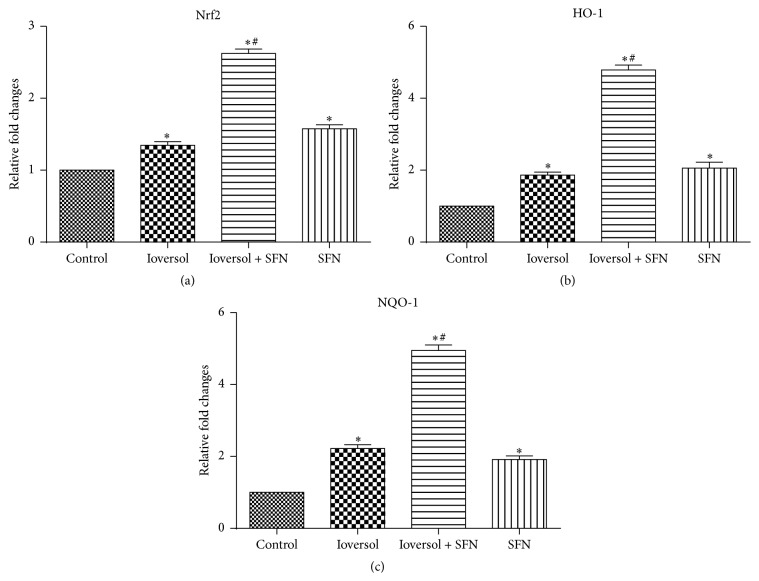
SFN increased the expression levels of Nrf2, HO-1, and NQO-1 in HK2 cells after Ioversol exposure. (a) The relative expression of Nrf2; (b) the relative expression of HO-1; and (c) the relative expression of NQO-1. ^*∗*^ *P* < 0.05 versus control group; ^#^ *P* < 0.05 versus Ioversol group.

The effects of SFN on the cellular ROS levels induced by H_2_O_2_ (500 *μ*mol/L) or 50 mg/mL Ioversol in vitro were measured using proximal tubule (HK2) cells. As shown in Figure 9, H_2_O_2_ or Ioversol significantly increased the ROS levels in HK2 cells. However, the H_2_O_2_- or Ioversol-induced ROS increase was significantly inhibited by pretreatment with SFN in a dose-dependent manner. SFN pretreatment protected against Ioversol-induced cytotoxicity in MTT assays. Moreover, to examine whether the renoprotective role of SFN in Ioversol-induced toxicity is associated with Nrf2 activation, the mRNA expression levels of Nrf2 and its target genes, NQO-1 and HO-1, were assessed using real-time PCR. As shown in Figure 10, the administration of SFN significantly increased Nrf2, NQO-1, and HO-1 gene expression in Ioversol-injured cells. The results also indicated that the renoprotective effect of SFN was associated with the Nrf2/HO-1 pathway.

### 3.7. SFN Exerts Its Renoprotective Role via the Activation of Nrf2 in HK2 Cells

**Figure 11:**
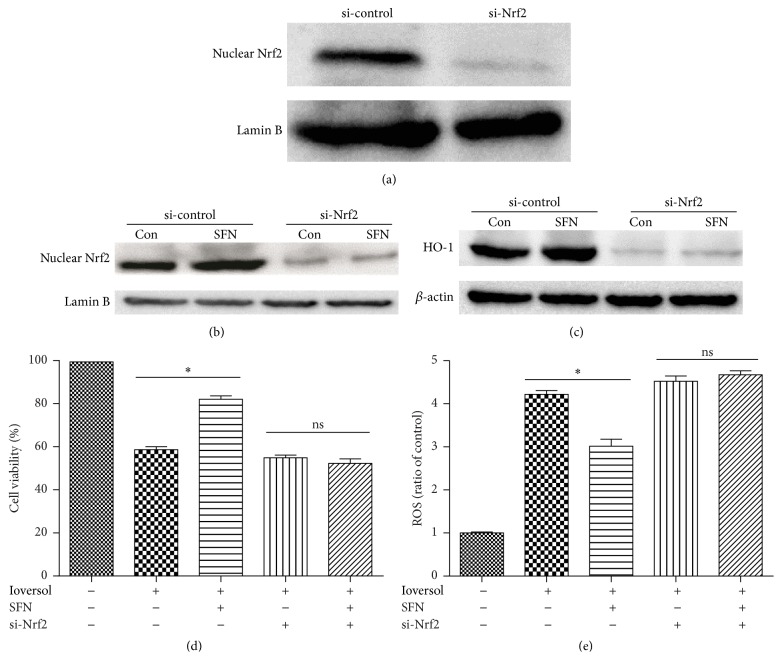
SFN exerts its renoprotective role via the activation of Nrf2 in HK2 cells. (a) Cells were treated for 48 h with control or Nrf2 siRNA (the transfection efficiency was measured by western blot analysis). (b) SFN did not increase the Nrf2 nuclear protein level in Nrf2-deficient cells. (c) SFN did not increase the HO-1 protein level in Nrf2-deficient cells. (d) SFN did not increase cell viability in Nrf2-deficient cells. (e) SFN did not decrease reactive oxygen species in Nrf2-deficient cells. ^*∗*^ *P* < 0.05; ns, no significance.

**Figure 12:**
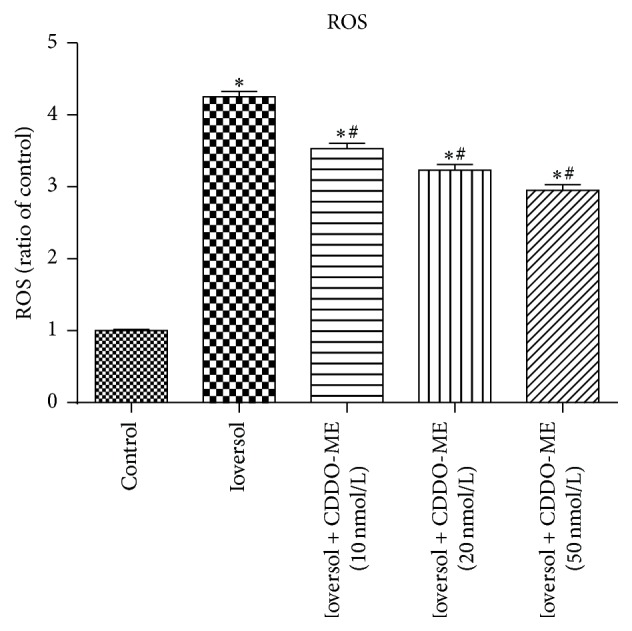
CDDO-ME (Nrf2 activator) attenuated reactive oxygen species in Ioversol-induced injury cells. ^*∗*^ *P* < 0.05, versus control group; ^#^ *P* < 0.05, versus Ioversol group.

To confirm the contribution of Nrf2 signaling to the renoprotective effect of SFN in HK2 cells after Ioversol-induced injury, the survival and viability of HK2 cells transiently transfected with Nrf2 siRNA were assessed after Ioversol treatment (Figure 11(a)). The efficiency of the Nrf2 siRNA-mediated knockdown of Nrf2 was measured by western blotting. Moreover, we investigated the effects of SFN on Nrf2 and HO-1 expression in Nrf2-deficient cells (Figures 11(b) and 11(c)). As shown in Figure 11(d), SFN did not increase the cell viability of Nrf2-deficient cells. However, the knockdown of Nrf2 increased the ROS level (Figure 11(e)). To confirm the role of Nrf2/HO-1 pathway in SFN mediated renoprotection, we also used Nrf2 activator CDDO-ME to investigate this effect. As listed in Figure 12, CDDO-ME also attenuated ROS in a dose-dependent manner. These data indicated that Nrf2 plays a crucial role in the renoprotective effects of SFN.

## 4. Discussion

CIN is a complex disorder with high incidence that may develop after exposure to iodinated contrast media [5]. Specifically, CIN occurs in approximately 5% of hospitalized patients who exhibit normal renal function prior to the injection of contrast medium and is responsible for 12% of all cases of acute renal failure in hospitals [23]. Moreover, the incidence of CIN is relatively high in high-risk patients, such as patients with diabetic nephropathy [24] or critically ill elderly patients [25]. Unfortunately, specific drugs that effectively treat CIN and can be widely used in the clinic are not available [5]. Thus, an effective strategy against CIN needs to be identified.

In the present study, we investigated the effects of SFN pretreatment before the administration of contrast media in rats. Our study indicated that the administration of contrast agent resulted in acute renal injury. Specifically, the rats in the Ioversol group exhibited deteriorated renal function and histopathological damage. Moreover, prominent increases in the renal tissue MDA and ROS levels were observed in the Ioversol group. In contrast, the administration of SFN before the induction of CIN effectively attenuated renal damage and decreased the MDA and ROS levels in the Ioversol + SFN group. In addition, our results also indicated that SFN may exert its renoprotective effect by increasing the expression of several antioxidant genes (Nrf2, HO-1, and NQO-1). SFN also enhanced Nrf2 protein nuclear translocation and increased the HO-1 and NQO-1 protein levels. In vivo, SFN increased cell viability and promoted the expression of Nrf2, HO-1, and NQO-1 in the context of Ioversol-induced injury.

The exact pathophysiology of CIN is not completely understood, but oxidative stress is generally recognized to play a critical role in the development of CIN. Exposure to contrast media results in renal medullary hypoxia and the generation of excessive levels of ROS [26, 27]. The ROS imbalance causes lipid peroxidation and changes in antioxidant enzyme activities, thus resulting in cytotoxic damage. Lipid peroxidation is generally measured on the basis of the production of MDA, which is an indicator of oxidative damage [27]. In this study, an increase in the MDA level was detected in the renal tissues of the Ioversol group, which indicated that contrast media caused oxidative damage, as previously reported [7]. Moreover, the decreased SOD activities in the renal tissues of the Ioversol group also suggested the presence of oxidative damage in the CIN rats. As shown in Figure 3, pretreatment with SFN decreased the renal MDA levels and increased the antioxidant enzyme SOD activities to protect against oxidative damage in CIN rats. In addition, in vitro pretreatment with SFN directly scavenged ROS in HK2 cells. Thus, the renoprotective effect of SFN can be attributed to the direct removal of excess ROS. Consistently with our findings, the antioxidant effects of SFN have also been reported in previous studies [28, 29]. Moreover, SFN also increased the viability of Ioversol-injured HK2 cells.

Cumulative evidence has suggested that the Nrf2 signaling pathway regulates many adaptive cytoprotective responses to counteract tissue damage caused by various environmental toxicants [17, 30, 31]. Upon activation, Nrf2 translocates to the nucleus and regulates the transcriptional activation of its target genes [32]. Previous studies have reported that SFN plays a crucial role in protecting against damage due to oxidative stress in different organs, such as the liver, skin, and heart [33, 34].

Growing evidence indicates that Nrf2 has a renoprotective role [35–37]. In the present study, pretreatment with SFN also contributed to the activation of Nrf2 and its target genes (HO-1 and NQO-1), as shown in Figure 3. Several studies have also indicated that SFN can activate the Nrf2 signaling pathway and suppress oxidative stress. In neural crest cells exposed to ethanol, SFN treatment neutralizes ROS by activating the Nrf2-mediated antioxidant response [29]. SFN also protects against cisplatin-induced nephrotoxicity by activating Nrf2 [38]. Furthermore, SFN has been demonstrated to prevent renal antioxidant imbalances in renal ischemic/reperfusion injury [39]. The activation of Nrf2 was also observed in western blot and immunohistochemical analyses, and SFN was shown to stabilize Nrf2. In Nrf2-deficient cells, SFN did not play a renoprotective role, which indicated that Nrf2 is important in the defense against oxidative damage.

Moreover, SFN administration also activated HO-1 and NQO-1 in the Ioversol + SFN group. Heme oxygenases (HOs) are enzymes that catalyze the degradation of heme. After degradation, heme is transformed into carbon monoxide (CO), free iron, and biliverdin. The different end products of heme catabolism have been reported to have antioxidant properties [40]. Three isoforms of heme oxygenase have been identified, namely, an inducible HO-1, a constitutive HO-2, and a catalytically inactive HO-3. Mounting evidence reveals that HO-1 is important in maintaining antioxidant and oxidant homeostasis in various diseases, such as radiation-induced injury, severe sepsis, and acute kidney injury [41]. In the present study, pretreatment with SFN increased the expression of HO-1 and attenuated kidney damage in CIN rats.

## 5. Conclusions

Our study demonstrated that SFN ameliorates CIN, as measured by renal function and kidney pathology. Moreover, SFN increased Nrf2 nuclear translation, the HO-1 protein level, cell viability, and expression of Nrf2, HO-1, and NQO-1 in Ioversol-injured HK2 cells.

These beneficial effects are mainly due to the improved antioxidant defense in the kidney and the activation of the Nrf2 pathway. Thus, SFN may be a valuable drug in the prevention of CIN; however, further experiments and randomized clinical trials are needed to determine its protective role.

## Figures and Tables

**Table 1:** Primers for selected genes.

Gene	Sense	Antisense
Nrf2	5′-GAGAATTCCTCCCAATTCAGC-3′	5′-TTTGGGAATGTGGGCAAC-3′
HO-1	5′-CTAAGACCGCCTTCCTGCT-3′	5′-TGTCTGTGAGGGACTCTGGTC-3′
NQO1	5′-TGACAAGGGTCCTTTCCAGA-3′	5′-CACCCTGCAGAGAGTACATGG-3′
*β*-actin	5′-CTGAACCCCAAAGCCAAC-3′	5′-CACCATCACCAGAGTCCATCAC-3′
